# Phytoestrogen Agathisflavone Ameliorates Neuroinflammation-Induced by LPS and IL-1β and Protects Neurons in Cocultures of Glia/Neurons

**DOI:** 10.3390/biom10040562

**Published:** 2020-04-07

**Authors:** Monique Marylin Alves de Almeida, Cleide dos Santos Souza, Naiara Silva Dourado, Alessandra Bispo da Silva, Rafael Short Ferreira, Jorge Mauricio David, Juceni Pereira David, Maria de Fátima Dias Costa, Victor Diógenes Amaral da Silva, Arthur Morgan Butt, Silvia Lima Costa

**Affiliations:** 1 Laboratory of Neurochemistry and Cellular Biology, Department of Biophysics and Biochemistry, Institute of Health Sciences, Federal University of Bahia, Salvador-Bahia 40.110-100, Brazil; moniquemarylin27@gmail.com (M.M.A.d.A.); souzacs14@gmail.com (C.d.S.S.); naiaradourado@hotmail.com (N.S.D.); alebispo19@gmail.com (A.B.d.S.); rafael_short@hotmail.com (R.S.F.); fatima@ufba.br (M.d.F.D.C.); vdsilva@ufba.br (V.D.A.d.S.); 2 INCT—Sheffield Institute of Translational Neuroscience (SITraN), The University of Sheffield, Sheffield S10 2HQ, UK; 3 Department of General and Inorganic Chemistry, Institute of Chemistry, Federal University of Bahia, Salvador-Bahia 40.170-115, Brazil; jmdavid@ufba.br; 4 Department of Medication, Faculty of Pharmacy, Federal University of Bahia, Salvador-Bahia 40.170-115, Brazil; juceni@ufba.br; 5 School of Pharmacy and Biomedical Science, University of Portsmouth, Portsmouth PO1 2DT, UK

**Keywords:** flavonoids, agathisflavone, neuroprotection, anti-neuroinflammation

## Abstract

Inflammation and oxidative stress are common aspects of most neurodegenerative diseases in the central nervous system. In this context, microglia and astrocytes are central to mediating the balance between neuroprotective and neurodestructive mechanisms. Flavonoids have potent anti-inflammatory and antioxidant properties. Here, we have examined the anti-inflammatory and neuroprotective potential of the flavonoid agathisflavone (FAB), which is derived from the Brazilian plant *Poincianella pyramidalis*, in in vitro models of neuroinflammation. Cocultures of neurons/glial cells were exposed to lipopolysaccharide (LPS, 1 µg/mL) or interleukin (IL)-1β (10 ng/mL) for 24 h and treated with FAB (0.1 and 1 µM, 24 h). FAB displayed a significant neuroprotective effect, as measured by nitric oxide (NO) production, Fluoro-Jade B (FJ-B) staining, and immunocytochemistry (ICC) for the neuronal marker β-tubulin and the cell death marker caspase-3, preserving neuronal soma and increasing neurite outgrowth. FAB significantly decreased the LPS-induced microglial proliferation, identified by ICC for Iba-1/bromodeoxyuridine (BrdU) and CD68 (microglia M1 profile marker). In contrast, FAB had no apparent effect on astrocytes, as determined by ICC for glial fibrillary acidic protein (GFAP). Furthermore, FAB protected against the cytodestructive and proinflammatory effects of IL-1β, a key cytokine that is released by activated microglia and astrocytes, and ICC showed that combined treatment of FAB with α and β estrogen receptor antagonists did not affect NF-κB expression. In addition, qPCR analysis demonstrated that FAB decreased the expression of proinflammatory molecules TNF-α, IL-1β, and connexins CCL5 and CCL2, as well as increased the expression of the regulatory molecule IL-10. Together, these findings indicate that FAB has a significant neuroprotective and anti-inflammatory effect in vitro, which may be considered as an adjuvant for the treatment of neurodegenerative diseases.

## 1. Introduction

The main players of the inflammatory response in the central nervous system (CNS) are microglia and astrocytes, which, once activated to a proinflammatory profile, begin to release different inflammatory mediators, such as cytokines, chemokines, and reactive oxygen and nitrogen species [1]. This sustained neuroinflammatory stimulus may contribute to neuronal death [2] and, therefore, to the pathogenesis of neurodegenerative diseases, including Multiple Sclerosis (MS), Alzheimer’s Disease (AD), and Parkinson’s Disease (PD) [3]. Drugs that display anti-inflammatory and antioxidant activity have considerable general potential for new therapies for these diseases.

As surveillant cells, microglia present a highly plastic phenotype [4]. Once a stressor is recognized, microglia rapidly shift to an activated state, which leads to changes in their morphology and molecular profile [5]. Activated microglia can be recognized as having an M1 phenotype (classically activated or proinflammatory) or M2 phenotype (alternatively activated or anti-inflammatory). Classically activated microglia (M1) can be characterized by the expression of MHC class II molecules and proinflammatory molecules, such as TNF-α, interleukin (IL)-β, IL-6, IL-18, nitric oxide, CCL2, and CCL5 [6]. Alternatively, activated microglia (M2) help to recover tissue homeostasis and express anti-inflammatory cytokines, such as arginase, IL-10, and TGF-β [7].

Flavonoids are natural products derived from plants that have been shown to exert potent anti-inflammatory, antioxidant, and immunomodulatory effects. Many studies suggest a correlation between flavonoid intake in the diet and a reduction in levels of dementia, in addition to its benefits to memory and the learning process [8,9]. Flavonoids modulate inflammatory responses involved in neurodegenerative diseases through the reduction of the expression of proinflammatory cytokines, including IL-6, TNF-α, IL-1β, and COX-2 [10,11]. In the CNS, the anti-inflammatory effects of flavonoids have been related to the control of astrogliosis and microgliosis [12,13]. Therefore, flavonoids are regarded as potential therapeutic agents for controlling inflammatory processes involved in neurodegenerative diseases [14].

In this study, we have investigated the neuroprotective and immunomodulatory effects of the phytoestrogen agathisflavone (FAB), which has known activity upon activation of estrogen receptors (ERs). FAB is a biflavonoid and, in this study, was extracted from *Poincianella pyramidalis* (Tul.), an endemic plant common in northeastern Brazil. We used an in vitro model of neuroinflammation, in which cocultures of neurons and glial cells were exposed to lipopolysaccharide (LPS) and IL-1β; LPS is a component of the Gram-negative bacteria cell membrane known to activate microglia and promote secondary neuronal damage [15], whereas IL-1β is an important cytokine responsible for the activation of the M1 proinflammatory profile in microglia [16]. The results demonstrated that FAB displayed a significant cytoprotective effect on neurons and an immunomodulatory effect on microglia, but these effects were not mediated via estrogen receptors in this model. 

## 2. Methods

### 2.1. Neuron–Glial Cell Cocultures

Cell cultures were obtained from cerebral hemispheres from Wistar rats. The animals were provided by the Department of Physiology of the Institute of Health Sciences of the Federal University of Bahia (Salvador, BA, Brazil). Primary cultures of glial cells were obtained from Wistar rats as described in our previous work [12]. Cerebral hemispheres from postnatal Wistar rats at the age of one to two days old were isolated aseptically and the meninges were mechanically removed. The cortex was dissociated mechanically and suspended in DMEM HAM F12 medium (Gibco^®^, Life Technologies, Burlington, ONT, Canada, 12500-062), supplemented with 2 mM l-glutamine, 0.011 g/L pyruvate, 10% FBS, 3.6 g/L Hepes, 33 mM glucose (Cultilab, Campinas-SP, Brazil), 100 IU/mL penicillin G, and 100 µg/mL streptomycin, and cultured in 100 mm Ø plates in a humidified atmosphere with 5% CO_2_ at 37 °C. Culture medium was changed every 2 days and cells were cultured for 15 days. Cells were then washed 3× with phosphate-buffered saline (PBS), detached with trypsin (Trypsin EDTA, Sigma Aldrich, Saint Luis, MO, USA, 9002077), plated at a density of 1 × 10^5^ cell/cm^2^, and maintained in culture for 72 h. After incubation, neurons obtained from cerebral hemispheres of 14–16-day-old Wistar rat embryos, using the same method described above for glial isolation, were suspended in supplemented DMEM/HAM F12 (Gibco^®^, Life Technologies, Carlsbad, CA-USA, 12500-062), and seeded at half the number of glial cells (5 × 10^4^ cells/cm^2^) onto the astroglial monolayer. Cells were incubated in a humidified atmosphere with 5% CO_2_ at 37 °C for 8 days in vitro (DIV), when treatments were performed.

### 2.2. Agents and Treatments

Agathisflavone (FAB) was extracted from *Poincianella pyramidalis* (Tul.) as previously described [17], stored at 100 mM in dimethyl sulfoxide (DMSO, Sigma Aldrich, Saint Luis, MO, USA, 472301), and kept out of light at −20 °C until use. For the experiments, FAB was diluted in culture medium to make a final concentration of 0.1 or 1 µM in the neuron–glial cocultures; control cultures were treated with DMSO, the vehicle of dilution of FAB. To induce neuroinflammation, at 26 DIV, cocultures were treated for 24 h with LPS (1 µg/mL; Sigma Aldrich, Saint Luis, MO, USA, L2880) or IL-1β (10 ng/mL; R&D Systems, Minneapolis, MN, USA, 501-RL-010); then, the medium was removed and replaced with medium containing just agathisflavone (0.1 or 1 µM) or vehicle, and cultures were analyzed after 24 h. To assess whether the effects of agathisflavone were mediated through ERs, neuron–glial cocultures were treated with the selective ER-α antagonist MPP dihydrochloride at 10 nM (1,3-bis(4-hydroxyphenyl)-4-methyl-5-[4-(2-piperidinylethoxy)phenol]-1H-pyrazole dihydrochloride; Sigma Aldrich, Saint Luis, MO, USA, M7068) or the selective ER-β antagonist PHTPP at 1 μM (4-[2-phenyl-5,7-bis(trifluoromethyl) pyrazolo[1,5-a]pyrimidin-3-yl]phenol; Tocris, Bristol, UK, #2662); control cultures were treated with DMSO vehicle.

### 2.3. Immunocytochemistry

For immunocytochemistry, cells were washed with PBS three times and fixed with 4% paraformaldehyde for 15 min at room temperature (RT). Cultures were washed three times with PBS, incubated with 0.3% Triton X-100 in PBS (Sigma Aldrich, Saint Luis, MO, USA, 9002-93-1) for 5 min, and blocked by incubation with PBS containing 5% bovine serum albumin (BSA) (Sigma Aldrich, Saint Luis, MO, USA, A9418)) for 1 h. After blocking, samples were incubated with primary antibodies diluted in PBS containing 1% of BSA overnight. Cells were washed with PBS three times. Then, secondary antibodies were added to cells and incubated for 2 h. The cells were washed with PBS three more times and incubated with 5.0 μg/mL 4,6-diamidino-2-phenylindole (DAPI, Invitrogen - Molecular Probes, Eugene, OR-USA) for nuclear staining. Staining was visualized on a fluorescence microscope (Leica, Wetzlar-Germany, DFC7000). Images were captured with a 20× or 40× objective. The following primary antibodies were used at the indicated dilutions: anti-Tubulin β3 (mouse, 1:500; BioLegend, San Diego, CA, USA, 801201), anti-glial fibrillary acidic protein (GFAP) (rabbit, 1:300; DAKO, Glostrup-Denmark, Z0334), anti-Iba-1 (ionized calcium-binding adaptor molecule 1, rabbit, 1:200; Wako, Richmond, VA, USA, 019-19741), anti-CD68 (rat, 1:100; Abcam, ab53444), anti-active caspase-3 (rabbit, 1:300; Chemicon, ab3623), anti-neurofilament (1:400; Abcam, Cambridge, UK, AB24574), and anti-NF-κB-P50 (mouse, 1:200; Santa Cruz Biotechnology, Santa Cruz, CA, USA, SC8414). The following secondary antibodies were used at the indicated dilutions: Alexa Fluor 488-conjugated goat anti-mouse IgG (1:500; Molecular Probes, A11001), Alexa Fluor 594-conjugated goat anti-rabbit IgG (1:500; Invitrogen, Molecular Probes, Eugene, OR, USA, A11037), Alexa Fluor 555-conjugated goat anti-rat IgG (1:500; Invitrogen - Molecular Probes, Eugene, OR, USA, A21434), Alexa Fluor 488-conjugated goat anti-rabbit IgG (1:500; Invitrogen, Molecular Probes, Eugene, OR, USA, A11008), and Alexa Fluor 594-conjugated goat anti-mouse IgG (1:500, Invitrogen - Molecular Probes, Eugene, OR, USA). The quantification was performed by analyzing the total number of positive cells (per marker), divided by the total number of nuclei (DAPI positive) × 100. 

### 2.4. Bromodeoxyuridine Cell Proliferation Assay

Proliferation was evaluated using bromodeoxyuridine (BrdU) (Sigma Aldrich, Saint Luis, MO, USA). BrdU (10 µM) was added to the wells at the start of each treatment. Cells were fixed and DNA was denatured by treatment with denaturing solution (2 N HCl) for 20 min at room temperature. Mouse anti-BrdU monoclonal antibody (1:200, Sigma Aldrich, Saint Luis, MO, USA, B8434) diluted in PBS was pipetted into the wells and allowed to incubate for 1 h. Unbound antibody was washed away and cells were incubated with Alexa Fluor 594 antibody specific for mouse IgG (1:500, Invitrogen - Molecular Probes, Eugene, OR, USA) diluted in PBS-T for 1 h under slow agitation at room temperature. After incubation, the cell nuclei were stained with DAPI (5 µg/mL) for 10 min at room temperature. All reagents were used in accordance with the manufacturer’s instructions. Experiments were performed in triplicate. Thereafter, cells were analyzed using a fluorescence microscope (Leica, Wetzlar-Germany, DFC7000). Quantification was analyzed with ImageJ 1.33u (Bethesda, MD, USA).

### 2.5. Fluoro-Jade B (FJ-B) Staining

FJ-B staining was used to investigate neuronal death. Neuron–glial cocultures were cultivated in 96-well black plates (1.5 × 10^4^ cells/cm^2^) and were treated with DMSO, LPS (1 µg/mL), or IL-1β in the corresponding wells for 24 h. Then, the medium was removed and replaced with medium containing agathisflavone 0.1 and 1 µM or IL-1 receptor antagonist (raIL-1, Sigma Aldrich, Saint Luis-MO, USA, 1 μg/mL) and kept for more than 24 h. After treatment, the cultures were fixed in ethanol at 4 °C for 10 min. Cultures were washed three times with PBS and then incubated with 0.3% Triton X-100 in PBS (Sigma Aldrich, Saint Luis, MO, USA) for 10 min. After washing in PBS three times, cells were incubated with 0.001% Fluoro-Jade B in PBS for 30 min at RT, under agitation and protection from light. After incubation, the cells were washed three times with PBS, incubated for 5 min at RT in the dark with 5 µg/mL DAPI for nuclear staining, and then washed three times with PBS. Analyses were performed on a spectrophotometer (Varioskan™ Flash Multimode Reader, Thermo Plate, Thermo Fisher Scientific, Inc., Vantaa, Finland), and the fluorescence intensity of each sample was measured at 480 nm for Fluoro-Jade B and 350 nm for DAPI. The values of absorbance of Fluoro-Jade B of each well were normalized to the DAPI absorbance in the same well.

### 2.6. RNA Isolation and cDNA Synthesis

Total RNA was isolated from primary cultures of rat microglia with Trizol^®^ reagent according to the manufacturer’s specifications. Afterwards, 1 × 10^4^ cells/cm^2^ were seeded in 60 mm plates and then treated for 24 h with 1 µg/mL LPS or IL-1β, 0.1 and 1 µM of FAB, or the combination of LPS and FAB. The samples were stored at −80 °C until the time of the analysis. The concentration and purity of RNA were determined by spectrophotometric analysis using a nanospectrum Kasvi (KASVI, Sao Jose dos Pinhais, PR, Brazil, K23-0002). DNA contaminants were removed by treating the RNA samples with DNase using the Ambion DNA-free kit (cat# AM1906, Invitrogen™, Life Technologies™, Carlsbad, CA, USA). For cDNA synthesis, SuperScript^®^ VILO™MasterMix (cat# MAN0004286, Invitrogen™, Life Technologies, Carlsbad, CA, USA) was used in a 20 µL reaction with a concentration of 2.5 µg of total RNA.

### 2.7. Quantitative PCR (qPCR)

Quantitative real-time PCR was performed using Taqman^®^ Gene Expression Assays (Applied Biosystems, CA, USA) containing two primers to amplify the sequence of interest, a specific Taqman^®^ MGB probe, and TaqMan Universal Master Mix II with UNG (catalogue# 4440038 Invitrogen, Life Technologies™, Carlsbad, CA, USA). The assays corresponding to the genes quantified in this study were IL1B (Rn00580432_m1), TNF Loc1036 (Rn01525859_m1), Nos2 (Rn00561646_m1), IL6 (Rn01410330_m1), CCL2 (Rn00580555_m1), CCL5 (Rn00579590_m1_m1), IL10 (Rn01483988_m1), ARG (Rn00691090_m1), and TGFB (Rn00572010_m1). Real-time PCR was performed using the Quant Studio 7 Flex™ Real-Time PCR System (Applied Biosystems™ by Life technology, Carlsbad, CA, USA). The thermocycling conditions were performed according to the manufacturer’s specifications. The actin beta (Actb) (Rn00667869_m1) and Hypoxanthine Phosphoribosyl Transferase 1 (HPRT1) (Rn01527840_m1) targets were used as reference genes (endogenous controls) for normalization of gene expression data. Data were analyzed using the 2^−ΔΔCt^ method. The results represent the average of 3 independent experiments. 

### 2.8. NO Production

Nitric oxide (NO) production was assessed as sodium nitrite (NaNO_2_^−^) accumulation in the culture medium using a colorimetric test based on the Griess reagent (Wang et al., 2002). Samples (50 µL) were collected after LPS damage and/or 24 h treatment with 0.1 and 1 µM of FAB. Equal volumes of culture medium and Griess reagent (1% sulfanilamide, 0.1% *N*-(1-naphthyl) ethylenediamine dihydrochloride, and 2% phosphoric acid Sigma Aldrich, Saint Luis, MO, USA) were mixed. The mixture was incubated for 10 min at room temperature and then the absorbance at 550 nm was measured using a microplate reader (Varioskan™ Flash Multimode Reader, Thermo Plate, Thermo Fisher Scientific, Inc., Vantaa-Finland). The concentrations of nitrite in the samples were determined based on a sodium nitrite standard curve (1.26–100 mmol/L NaNO_2_). Three independent experiments were performed.

### 2.9. Statistical Analyses

Statistical analyses were performed using GraphPad Prism 5. We first analyzed whether the values came from a Gaussian distribution. Kruskal–Wallis followed by Dunn’s multiple comparison test was performed for non-normal samples; for normal samples, we performed one- or two-way analysis of variance (ANOVA) followed by Tukey’s or Bonferroni’s post-tests, respectively. Confidence intervals were defined at a 95% confidence level (*p* < 0.05 was considered to be statistically significant). Fold change was calculated by dividing the average (mean) value of the experimental group by that of the control group. In all figures, error bars represent SEM of at least 3 independent experiments.

### 2.10. Ethics Approval

All experiments were performed in accordance with the local Ethical Committee for Animal Experimentation of the Health Sciences Institute (Protocol No. 027/2012).

## 3. Results

### 3.1. Agathisflavone (FAB) Protects Against LPS-Induced Neuroinflammatory Damage 

**Figure 1:**
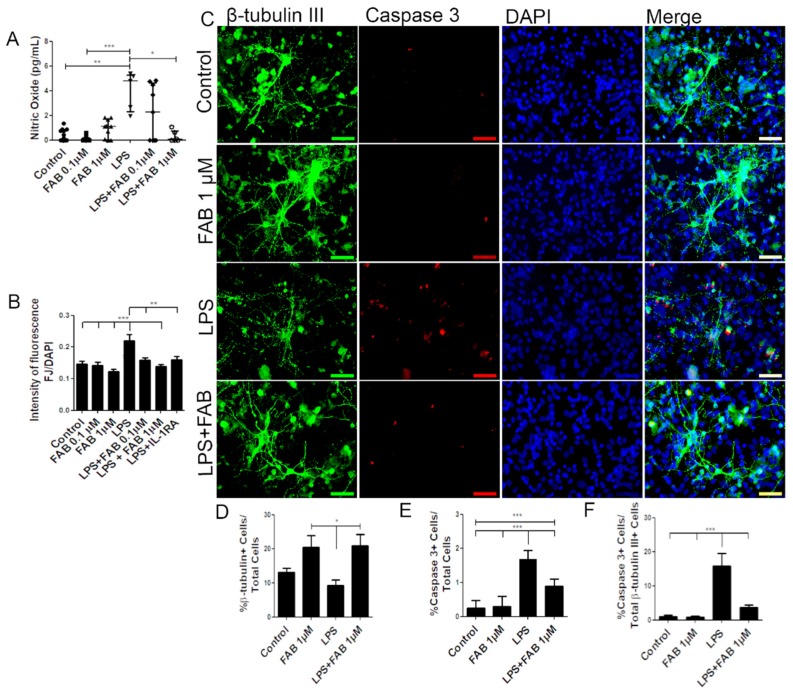
Agathisflavone (FAB) protects neurons against lipopolysaccharide (LPS)-induced neuroinflammatory damage. Cells were incubated with 0.01% of vehicle (dimethyl sulfoxide, DMSO) or treated with 1 µg LPS, 0.1 and/or 1 µM of FAB, or during 24 h of damage by LPS followed by a further 24 h using 0.1 and/or 1 µM of FAB. (**A**) Boxplot graph showing the Griess reaction for NO determination; results are expressed as NaNO_2_; values are expressed as the median and tested for significance by the Kruskal–Wallis test followed by Dunn’s test. (**B**) Bar graph of the proportion of neurons undergoing degeneration measured by the Fluoro-Jade B (FJ-B) fluorescence intensity/4,6-diamidino-2-phenylindole (DAPI) fluorescence intensity stain; values are expressed as the mean ± SEM (*n* = 3) and were tested for significance by one-way ANOVA. (**C**) Representative photomicrographs of immunocytochemistry (ICC) for detection of the apoptotic marker caspase-3 (red), counterstained with the neuronal marker β-Tubulin III+ (green) and nuclei marked with DAPI (blue) by fluorescence microscopy; scale bar: 50 µm. (**D**–**F**). Bar graphs showing the percentage of βTubIII+, caspase-3^+^ cells, and βTubIII+/caspase-3^+^ cells in each condition; values are expressed as the mean ± SEM (*n* = 3) and were tested for significance by one-way ANOVA. *** *p* < 0.001, ** *p* < 0.01, and * *p* < 0.05.

In order to investigate the neuroprotective potential of FAB against neuroinflammatory damage, we first used LPS, a component of Gram-negative bacteria cell membrane known to activate microglia and astrocytes and to promote secondary neuronal damage [15]. Neuron–glial cocultures were exposed to 1 µg/mL of LPS for 24 h and then treated with FAB (0.1–1 µM) for 24 h. First, we evaluated production of nitric oxide (NO), which is neurocytotoxic and is produced by activated M1 microglia [18]. Treatment with LPS induced an increase in NO compared with control, and this was significantly reduced to control levels by treatment with 1 µM FAB (Figure 1A). In addition, FJ-B, a measure of neuronal degeneration, was significantly increased after LPS treatment compared with controls, and this was blocked by FAB treatment (Figure 1B). To unequivocally determine that FAB was neuroprotective, we used immunolabeling for the neuronal marker β-tubulin III and the cell apoptosis marker caspase-3 (Figure 1C). The results showed that FAB induced an increase in the number of neurons (20.5%) in relation to control cultures (13.2%) and was neuroprotective against LPS (Figure 1D). Cultures exposed to LPS showed a significant increase in cell death overall, as determined by the number of caspase-3^+^ cells, and this was significantly reduced by treatment with FAB (1 µM) (Figure 1E). In neurons specifically, costaining of β-tubulin III and caspase-3 demonstrated a significant increase in neuronal cell death in LPS (15.9%), which was almost completely blocked by FAB treatment, where the percentage of caspase-3^+^ neurons decreased to 3.75% (Figure 1F). 

### 3.2. Agathisflavone (FAB) Preserves Neurons and Decreases Microglial Proliferation after LPS-Induced Neuroinflammatory Damage 

**Figure 2:**
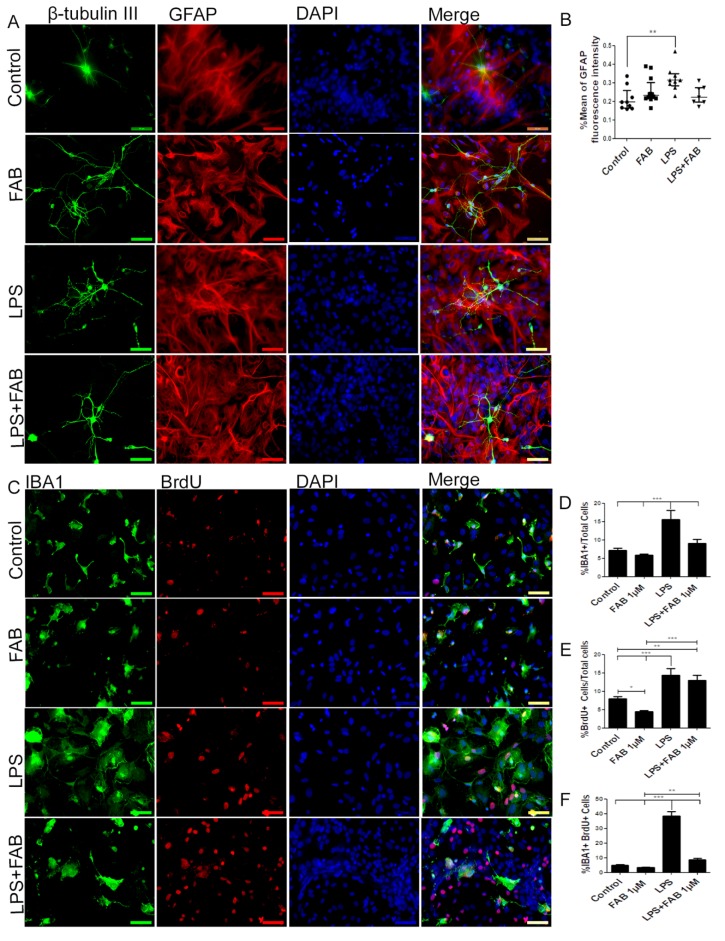
Agathisflavone preserves neuronal morphology and decreases microgliosis. (**A**) Representative photomicrographs of immunolabeling for the neuronal marker β-TubIII (green) and astrocyte marker glial fibrillary acidic protein (GFAP) (red); nuclei were counterstained with DAPI (blue); scale bar: 50 µm. Representative images were chosen from the field that better represented astrocyte–neuron proximity. (**B**) Boxplot graph showing GFAP immunofluorescent intensity (% mean); values are expressed as the median and were tested for significance using the Kruskal–Wallis test followed by Dunn’s test. (**C**) Analysis of cell proliferation by bromodeoxyuridine (BrdU) (red) incorporation into the DNA of microglia cells (Iba-1, green) exposed to 1 µM of FAB or in control conditions (0.1% DMSO) 24 h after treatment. Cell nuclei were stained with DAPI (blue). Scale bar: 50 µm. (**D**–**F**) Bar graphs showing the total number of Iba-1^+^ cells, total BrdU^+^ cells, and BrdU^+^/Iba-1^+^ cells (microglial proliferation) expressed as a percentage of the total number of cells counted; values are expressed as the mean ± SEM (*n* = 3) and were tested for significance by one-way ANOVA. ** *p* < 0.01 and *** *p* < 0.001.

In order to characterize morphological changes in neurons, astrocytes, and microglia, as well as in astrocyte reactivity and microglial proliferation after inflammatory stimulus with LPS, immunocytochemical analyses were performed in cocultures of neurons and glial cells. After treatment with LPS, the neuronal dendrite network was lost, forming only irregular clusters with loss of the morphological pattern observed under control conditions (Figure 2A). In contrast, FAB treatment preserved neuronal integrity (Figure 2A), as shown by preservation of the neuronal cell body and enhancement of neurites. In addition, astrocytes retained their typical polygonal morphology in FAB, similar to that observed under control conditions (Figure 2A). Quantitation indicated that the mean GFAP fluorescence intensity was significantly increased in LPS, a characteristic of reactive astrocytes, whereas GFAP fluorescence intensity was not statistically different than controls in FAB + LPS (Figure 2B). 

Microglia were examined using immunolabeling for Iba-1, a general microglial marker that identifies both M1 and M2 phenotypes [19]. In controls, microglia had a process-bearing phenotype, which was also observed after FAB treatment alone (Figure 2C). In contrast, treatment with LPS clearly induced an increase in the proportion of cells with an amoeboid phenotype, which is characteristic of phagocytic M1 microglia (Figure 2D), as well as microglial proliferation determined by BrdU incorporation (Figure 2E). After treatment with FAB, there was an evident decrease in the number of amoeboid microglia (Figure 2C). Quantitation demonstrated that LPS treatment resulted in a doubling of microglial numbers (Figure 2D) and microglial proliferation (Figure 2F, Iba-1^+^/BrdU^+^ cells), and these were significantly decreased by FAB (Figure 2D,F). Interestingly, LPS induced an overall increase in cell proliferation in the cultures and this was not significantly affected by FAB (Figure 2E), an effect that might be related to neuronal and astroglial proliferation. 

### 3.3. Agathisflavone (FAB) Exerts an Anti-Inflammatory Effect against LPS in Microglia 

**Figure 3:**
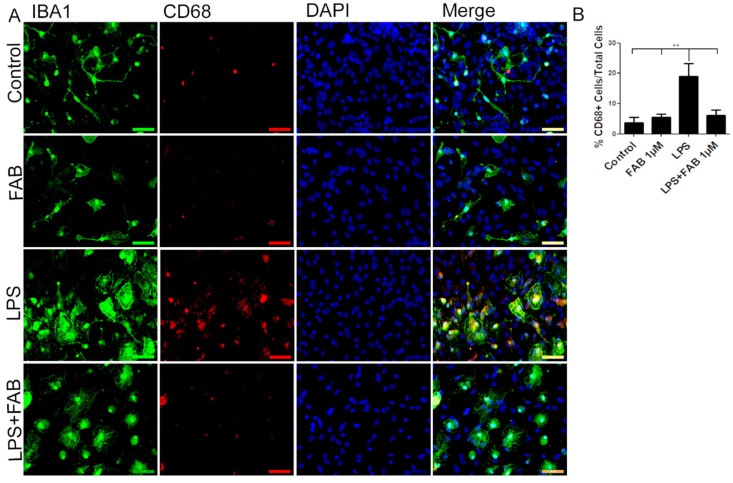
Agathisflavone exerts an anti-neuroinflammatory effect against damage induced by LPS. (**A**) Representative photomicrographs of immunocytochemistry for Iba-1 (green) and the M1 microglial marker CD68 (red) counterstained with DAPI (blue) in cocultures; scale bar: 50 µm. (**B**) Bar graph showing the percentage of CD68^+^/Iba-1^+^ cells in each treatment group; values are expressed as the mean ± SEM (*n* = 3) and were tested for significance by one-way ANOVA. ** *p* < 0.01.

In order to evaluate the immune profile of microglia in cocultures treated with LPS and after treatment with FAB, immunocytochemistry for Iba-1 (a general marker for microglia) and CD68, a M1 profile microglial/macrophage marker, was conducted (Figure 3). We observed a marked increase in the expression of CD68 in cells exposed to LPS compared with the control, and this was completely blocked by treatment with FAB (Figure 3A,B). There was no significant difference between the expression of CD68 in cells in control conditions and those treated with FAB. 

### 3.4. Agathisflavone (FAB) Protects Against IL-1β-Induced Neuroinflammatory Damage 

**Figure 4:**
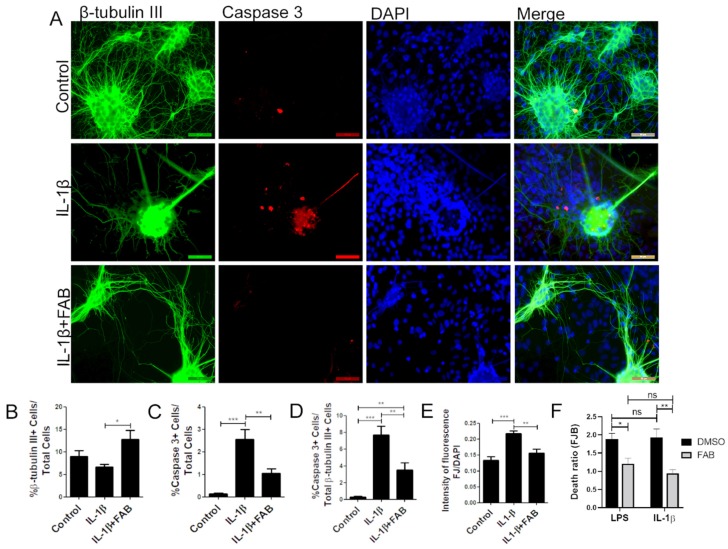
Agathisflavone protects neurons against neuroinflammation induced by interleukin (IL)-1β. (**A**) Representative photomicrographs of immunofluorescence labeling for the apoptotic marker caspase-3 (red), the neuronal marker β-TubIII^+^ (green), and nuclear stain DAPI (blue); scale bar: 50 µm. (**B**–**F**) Bar graphs showing the percentage of β-TubIII^+^, caspase-3^+^ cells, and βTubIII^+^/caspase-3^+^ cells in each condition (**B**–**D**), together with the proportion of neurons undergoing degeneration measured by the FJ-B/DAPI fluorescence intensity stain after IL-1β and FAB (**E**) and comparing FAB with LPS and IL-1β (**F**); values are expressed as the mean ± SEM (*n* = 3) and were tested for significance by two-way ANOVA followed by Bonferroni’s post-test. *** *p* < 0.001, ** *p* < 0.01, and * *p* < 0.05.

In order to analyze whether FAB also protects against neuroinflammation induced by IL-1β, neuron–glial cocultures were exposed to 10 ng/mL IL-1β for 24 h and then treated with FAB (1 µM) for 24 h. IL-1β is a proinflammatory cytokine highly expressed in neuroinflammatory conditions and an important immune mediator involved in a variety of cellular activities [20]. The same features were found regarding IL-1β compared to LPS, and FAB was able to preserve neuronal morphology (Figure 4A) and increase the number of β-tubulin-positive neurons compared with IL-1β treatment (Figure 4B). As with LPS, IL-1β increased the number caspase^+^ cells overall (Figure 4A,C) and specifically β-tubulin-positive neurons (Figure 4A,D) and FJ-B staining of necrotic cells (Figure 4E). Combined treatment with FAB significantly reduced the total cells in apoptosis (Figure 4C), as well as apoptotic neurons (Figure 4D) and necrosis (Figure 4E). Further analysis of Fluoro-Jade B (Figure 4F) showed no significant difference between LPS and IL-1β damage, which suggests that neuroprotection mediated by FAB might be related to the inhibition or control of the IL-1β signaling pathway. 

### 3.5. Agathisflavone (FAB) Preserves Neurons and Astrocytes and Decreases Microglia Proliferation after IL-1β-Induced Neuroinflammatory Damage 

**Figure 5:**
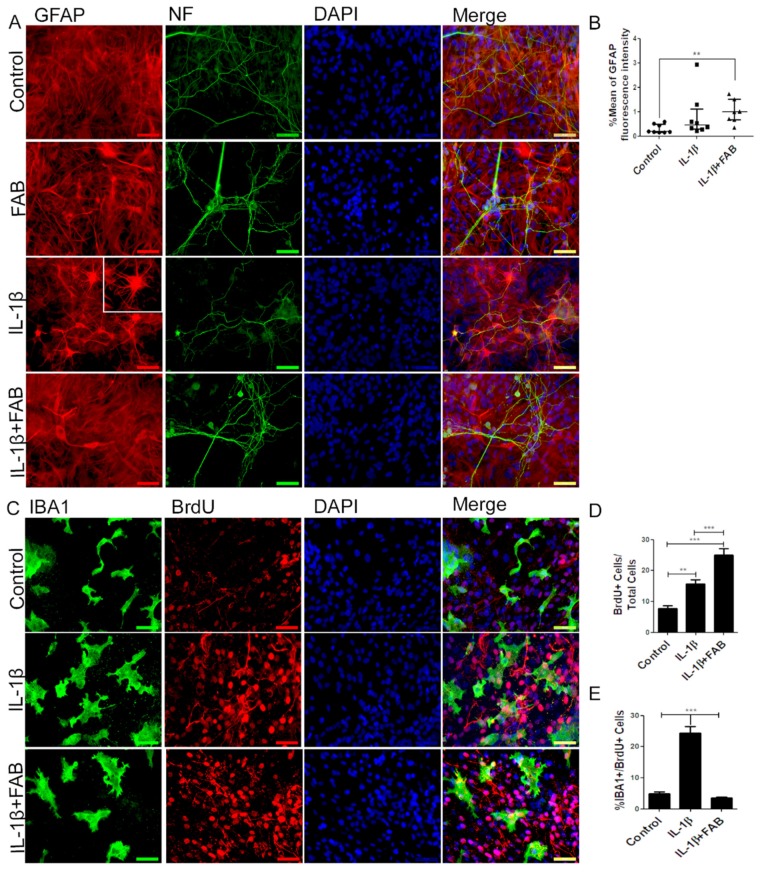
Agathisflavone preserves neuronal and astrocyte morphology, as well as decreases microglia proliferation. (**A**) Representative photomicrographs of immunocytochemistry for the astrocyte marker GFAP (red) and the axonal marker neurofilament (green), counterstained with the nuclear dye DAPI (blue); scale bar: 50 µm. (**B**) Boxplot graph showing GFAP fluorescent intensity; values are expressed as the median and were tested for significance using the Kruskal–Wallis test followed by Dunn’s test. (**C**) Analysis of cell proliferation by BrdU (red) incorporation into the DNA of microglia cells (Iba-1, green) exposed to 1 µM of agathisflavone or in control conditions (0.1% DMSO) 24 h after treatment, counterstained with the nuclear dye (blue); scale bar: 50 µm. (**D**,**E**) Bar graphs show the quantification of total BrdU^+^/DAPI cells (D) and Iba-1/BrdU^+^ microglia; values are expressed as the mean ± SEM (*n* = 3) and were tested for significance by one-way ANOVA. ** *p* < 0.01 and *** *p* < 0.001.

We next analyzed the effect of FAB after neuroinflammatory damage induced by IL-1β on axonal (Figure 5A) and astrocyte (Figure 5A) morphology and on GFAP fluorescence intensity (Figure 5B). In neurons, FAB exerts an apparent protective effect, as indicated by the maintenance of morphology and axonal integrity, which was severely disrupted in IL-1β (Figure 5A). Our findings showed that IL-1β did not alter GFAP fluorescence intensity but strongly altered astrocyte morphology, characterized by the presence of processes that extend from the cell body, apparently thinner and longer. In contrast, after treatment with FAB, the morphology of astrocytes remained as a monolayer of cells similar to the control, although there was no difference in GFAP fluorescence intensity. 

In order to investigate the anti-neuroinflammatory effect of FAB, we performed immunocytochemistry to analyze microglial proliferation and phenotype. After treatment with IL-1β, Iba-1 immunocytochemistry showed that microglia were much larger and presented a more amoeboid/phagocytic phenotype (Figure 5C). In addition, IL-1β markedly stimulated overall proliferation in cell cultures (Figure 5C,D) and specifically microglial proliferation (Figure 5C,E). Treatment with FAB did not have a marked effect on the amoeboid microglial morphology induced by IL-1β treatment (Figure 5C), but it completely inhibited the IL-1β-induced microglial proliferation (Figure 5E). Interestingly, the overall cellular proliferation in the cultures was increased by FAB treatment (24.9%, Figure 5D) in comparison with control (7.8%) and IL-1β damage (15.7%), which is consistent with the apparent increase in neurons and possibly astrocytes observed after FAB treatment (Figure 5A,B). 

**Figure 6:**
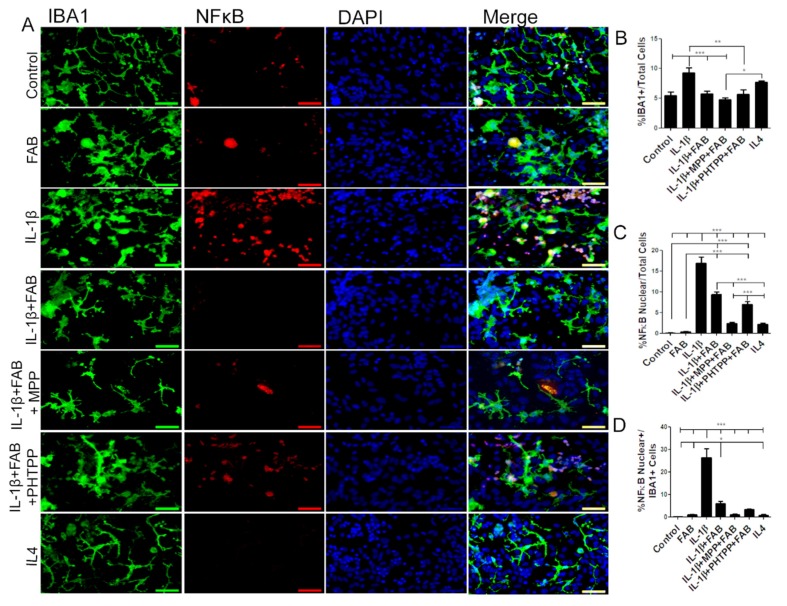
Agathisflavone protects against neuroinflammation induced by IL-1β. (**A**) Representative photomicrographs of immunocytochemistry for Iba-1 (green) and the M1 microglial marker NF-κB (red) counterstained with DAPI (blue) in cocultures; scale bar: 50 µm. B-D. Bar graphs showing total Iba-1^+^ microglia (**B**), total NF-κB+ cells (**C**), and NF-κB^+^/Iba-1^+^ microglia (**D**) in each treatment group, as indicated; values are expressed as the mean ± SEM (*n* = 3) and were tested for significance by one-way ANOVA. * *p* < 0.05, ** *p* < 0.01, and *** *p* < 0.001.

Microglia were examined further by double immunofluorescence labeling for Iba-1 and NF-κB, a proinflammatory profile marker (Figure 6). After neuroinflammatory stimulus induced by IL-1β, the total number of Iba-1^+^/NF-κB^+^ microglia was dramatically increased and this was significantly reduced by treatment with FAB (Figure 6A–C). Previous studies have indicated that FAB may act on estrogen receptors (ERα and ERβ) (12), and so we examined whether this may be the case for the immunomodulatory effect of FAB. Treatment with the ERα antagonist MPP or ERβ antagonist PHTPP did not inhibit the effects of FAB on IL-1β-induced changes in total Iba-1^+^ or NF-κB^+^/Iba^+^ microglia (Figure 6A–D, indicating that the anti-neuroinflammatory effect of FAB is not mediated via estrogen receptors. In addition, the effects of the anti-inflammatory cytokine IL-4 was tested as a positive anti-inflammatory control and there were differences neither in the morphology, number, and microglia expressing NF-κB nor in the total number of NF-κB cells regarding the control.

### 3.6. Agathisflavone (FAB) Positively Impacts Neuroinflammatory Gene Expression after LPS- and IL1-β-Induced Damage 

**Figure 7:**
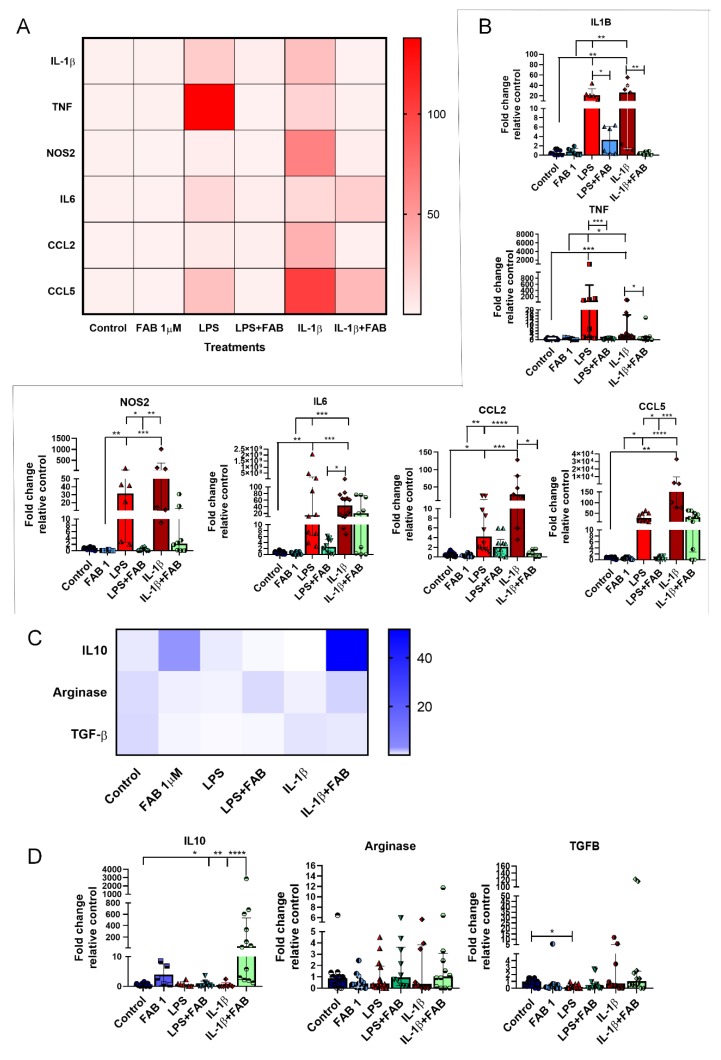
RT-qPCR of key inflammatory genes in neuron–glial cocultures exposed to LPS- or IL-1β-induced damage and treated with 1 µM FAB. (**A**) Heat map of inflammatory genes showed an important effect against proinflammatory molecules commonly implicated in most neurodegenerative diseases. (**B**) Scatter dot plot bar graphs showing quantitative data obtained from real-time qPCR of the proinflammatory genes shown on heat map; values are expressed as mean ± SEM or median ± interquatile range (IQR), and samples with Gaussian distribution were analyzed by one-way ANOVA followed by Tukey’s post hoc test, while nonparametric samples (scatter plot graph) were analyzed by the Kruskal–Wallis test followed by Dunn’s test. (**C**) Heat map of genes involved in the control of inflammation. (**D**) Scatter dot plot bar graphs show the expression of anti-inflammatory genes and respective differences among the treatments; values are expressed as the median ± IQR. Significant differences are indicated with * *p* < 0.05, ** *p* < 0.01, *** *p* < 0.001, and **** *p* < 0.0001.

Finally, we investigated whether the neuromodulatory and neuroprotective effects of FAB are associated with changes in expression of regulatory and inflammatory molecules (Figure 7). Once LPS or IL-1β binds to its specific receptor (Toll-like receptor 4 (TLR4) and IL1R, respectively), several signal transduction pathways are activated, which in turn lead to increased expression of inflammatory molecules [21,22], with important roles for IL-1β, TNF-α, IL-6, NOS2, CCL2, and CCL5 in neuronal death [22,23,24,25,26,27], while IL-10, arginase, and TGF-β are important regulatory molecules involved in the control of the inflammatory response and neuroprotection [11,19,28]. Both LPS and IL-1β induced an increase of mRNA expression of neuroinflammatory molecules IL-1β, TNF, and NOS2, as well as the expression of the chemokine CCL2, and these increases were almost completely blocked by FAB treatment (Figure 7A,B). In addition, LPS and IL-1β upregulated IL-6 and CCL5, but these were differentially affected by FAB, which only significantly reduced the effects of LPS on CCL5 (Figure 7A,B). Analysis of the regulatory molecules arginase, IL-10, and TGF-β indicated that FAB only significantly altered IL-10 in IL-1β treatment (Figure 7C,D). 

## 4. Discussion

Flavonoids are natural plant-derived compounds that have attracted considerable attention as potential treatments for neurodegenerative diseases because of their antioxidant [29,30] and immunomodulatory [31] activities. They are polyphenolic compounds synthesized in response to stress and found in fruits, seeds, grains, vegetables, flowers, wines and teas [32]. Biflavonoids are a specific class of flavonoids composed of a combination of C-C or C-O-C bonded flavonoid dimers [33] and have shown greater pharmaceutical efficiency than their respective monomers [34]. Here, using an established LPS and IL-1β model of neuroinflammation [35], we demonstrated that the biflavonoid agathisflavone (FAB) had potent neuroprotective and immunomodulatory effects in neuron–glial cocultures. A key action of FAB is to inhibit the proinflammatory function of microglia and direct them towards an anti-inflammatory M2-like phenotype. The results support FAB as a promising therapy for protecting against neurodegeneration and promoting tissue repair.

Current work using an in vivo model demonstrated that albino Swiss mice treated with high doses of agathisflavone did not show toxicity in any analyzed parameter (hematological, biochemical, histopathological, behavioral, as well as physiological) and presented LD50 larger than 2000 mg/kg, which is indicative of low toxicity [36]. A key finding of this study is that FAB is not toxic in both concentrations tested and has an important neuroprotective effect, in support of studies demonstrating neuroprotective effects of other biflavonoids, including amentoflavone, ginkgetin, and isoginkgetin, in oxidative-stress-induced and amyloid-β-peptide-induced cell death [37]. In the present study, LPS and IL-1β were shown to result in neuronal disruption and increased neuronal cell death, as measured by expression of caspase-3, which plays a pivotal role in apoptosis [38]. Notably, FAB decreased the number of caspase-3^+^ neurons and increased the overall number of neurons (β-Tubulin III^+^ cells) and BrdU^+^ cells, indicating that in addition to being neuroprotective, FAB has a neurogenic effect, as we have shown previously for FAB in murine pluripotent stem cells [39]. 

The neuroprotective effect of FAB is undoubtedly related to its inhibition of LPS- or IL-1β-induced microglial activation. It is known that microglial activation in the CNS is heterogeneous and often categorized into M1 and M2 phenotypes, which respectively have either cytotoxic or neuroprotective actions. Notably, dynamic changes in M1/M2 phenotypes have been associated with multiple neurodegenerative diseases, including AD, MS, stroke, and traumatic injury [40]. In general, M1 microglia predominate at the site of injury, and persistently activated M1 responses lead to neuronal loss. In contrast, the M2 phenotype is anti-inflammatory and is associated with the repair process. Hence, controlling the switch from M1 to M2 phenotype has considerable therapeutic benefit. LPS and IL-1β are potent inducers of the proinflammatory M1 microglial phenotype, characterized by the expression of the CD68 and NF-κB markers, as well as microglial proliferation (identified by BrdU incorporation) [41]. Our results demonstrate that, compared with the cells treated with LPS or IL-1β, FAB improved microglial morphology, reduced microglial proliferation (BrDU^+^ cells), and decreased the M1 markers CD68 and NF-κB. In addition, we demonstrated that LPS directly induced production of NO in the cocultures and that this was significantly reduced by FAB. At high concentrations, NO reversibly inhibits mitochondrial respiration by competing with O_2_ in cytochrome c oxidase and is neurotoxic [6,42,43,44,45,46]. Blockade of NO production by FAB is a likely mechanism by which it protects against LPS-induced neuronal death. 

An important finding is that FAB reduced the LPS- and IL-1β-induced expression of proinflammatory molecules, such as IL-1β, TNF, IL-6, CCL2, and CCL5, and increased the expression of regulatory molecule IL-10, protecting cortical neurons from inflammation. Comparable results were found in another study published by our group using the same approach (coculture of neurons and glia), which showed that after excitotoxicity induced by glutamate, FAB was able to reduce cell death induced by glutamate through reducing the expression of proinflammatory cytokines, including TNF, IL-1β, and IL6, and increasing the expression of IL10 and arginase 1 [12]. Astrocytes are also involved in the regulation of immune responses in neurodegenerative diseases [47]. Astrocytes respond to most CNS insults by reactive astrogliosis, characterized by cellular hypertrophy and increased GFAP expression, often associated with cell proliferation [48]. Under continuous stimulation, reactive astrocytes can potentiate neuroinflammation, as well as compromise homeostasis and synaptic function [48]. Here, we demonstrated that LPS and IL-1β altered astrocyte morphology, characteristic of astrocytic reactivity, and this was controlled by FAB. 

LPS is recognized by TLR4, which is highly present in the plasma membrane of microglia and astrocytes [49], which activates several signal transduction pathways and, at the end, causes NF-κB activation and results in raised transcription of genes encoding proinflammatory cytokines, especially the IL-1 family of cytokines [50], chemokines, and inducible enzymes that lead to neuroinflammation and consequent neuronal death. Once activated, NF-κB may regulate the expression of immune and inflammatory genes [51]. Interestingly, FAB treatment caused an overall decrease in NF-κB and this was even more pronounced in microglia. It is known that in neurons, NF-κB promotes survival and plasticity, contrary to its effects in glial cells, which play an important role in promoting inflammation and leading to neuronal damage [52].

Although previous studies [12] have shown that FAB acts via estrogen signaling to improve the neuroprotective properties of microglia, the immunomodulatory effect of FAB on NF-κB expression in the neuroinflammation model induced by IL-1β, however, was not mediated by estrogen receptors, agonists of which have proved to play a pivotal role in modulating microglial inflammatory response [53]. In addition, we demonstrated that neurodegeneration seen through Fluoro-jade B in LPS and IL-1β treatments was similar, as well as the neuroprotective effect of FAB, which suggests that FAB reduces neuronal death by a mechanism that seems to be related to the inhibition or control of the IL-1β signaling pathway. 

## 5. Conclusions

Overall, the major immunomodulatory effects on microglia and astrocytes are likely to be central to the neuroprotective action of FAB. Moreover, it may be a potential anti-inflammatory and neuroprotective agent to prevent and treat neuroinflammatory-related diseases.
